# Strangled rectal prolapse in young adults: A case report

**DOI:** 10.1016/j.amsu.2020.04.030

**Published:** 2020-05-11

**Authors:** Iyed Kraiem, Tarek Kellil, Mohamed Ali Chaouch, Ibtissem Korbi, Khadija Zouari

**Affiliations:** Department of Visceral Surgery, Fattouma Bourguiba Hospital, Monastir, Tunisia

**Keywords:** Rectal prolapse, Stranguled, Altemeir procedure, Emergent

## Abstract

**Background:**

Rectal prolapse (RP) is an uncommon perineal disease. It is defined as a complete protrusion or intussusception of the rectum through the anus. Strangulation of the RP is rare. This complication presents requires an emergent surgery. This case presentation aims to report the therapeutic management and results of this condition.

**Observation:**

A 29-year-old men, who consulted for a sudden, painful, irreducible rectal prolapse. At the anus, there was an irreducible, edematous, without signs of ischemia or necrosis rectal prolapse measuring 25*10 cm wide**.** The laboratory data showed a high white blood cell count and elevated C-reactive protein. After a failure of external manual reduction under general anesthesia, the patient underwent emergent surgery. The procedure consisted of a rectosigmoidectomy with coloanal anastomosis using a perineal approach according to the Altemeier technique associated to a diverting ileostomy. The postoperative follow-up was uneventful. The patient was discharged at post-operative day five.

**Conclusion:**

Strangulated RP is a rare complication. Altemeier procedure remains the intervention of choice in this situation.

## Highlights

• An uncommon perineal disease.
• Therapeutic surgical management and issues.
• Socio-psychological impact of this disease.

## 1 Introduction

Rectal prolapse (RP) is defined as a complete protrusion or intussusception of the rectum through the anus [1]. It concerns children aged between 1 and 3 years as well as the elderly [2]. Its occurrence for adults aged less than 30 years old is rare, as it is demonstrated by the lack of publications on the subject. Medication induced constipation in psychiatric patients and possible pelvic floor weakness in patients with previous pelvic surgery may be contributing factors to rectal prolapse. Strangulation of the RPis a rare complication that occurs in 2–4% of the cases [3,4]. This complication presents always an indication of urgent surgery. This case presentation aims to report the therapeutic management and results.

### 1.1 Case presentation

**Fig. 1:**
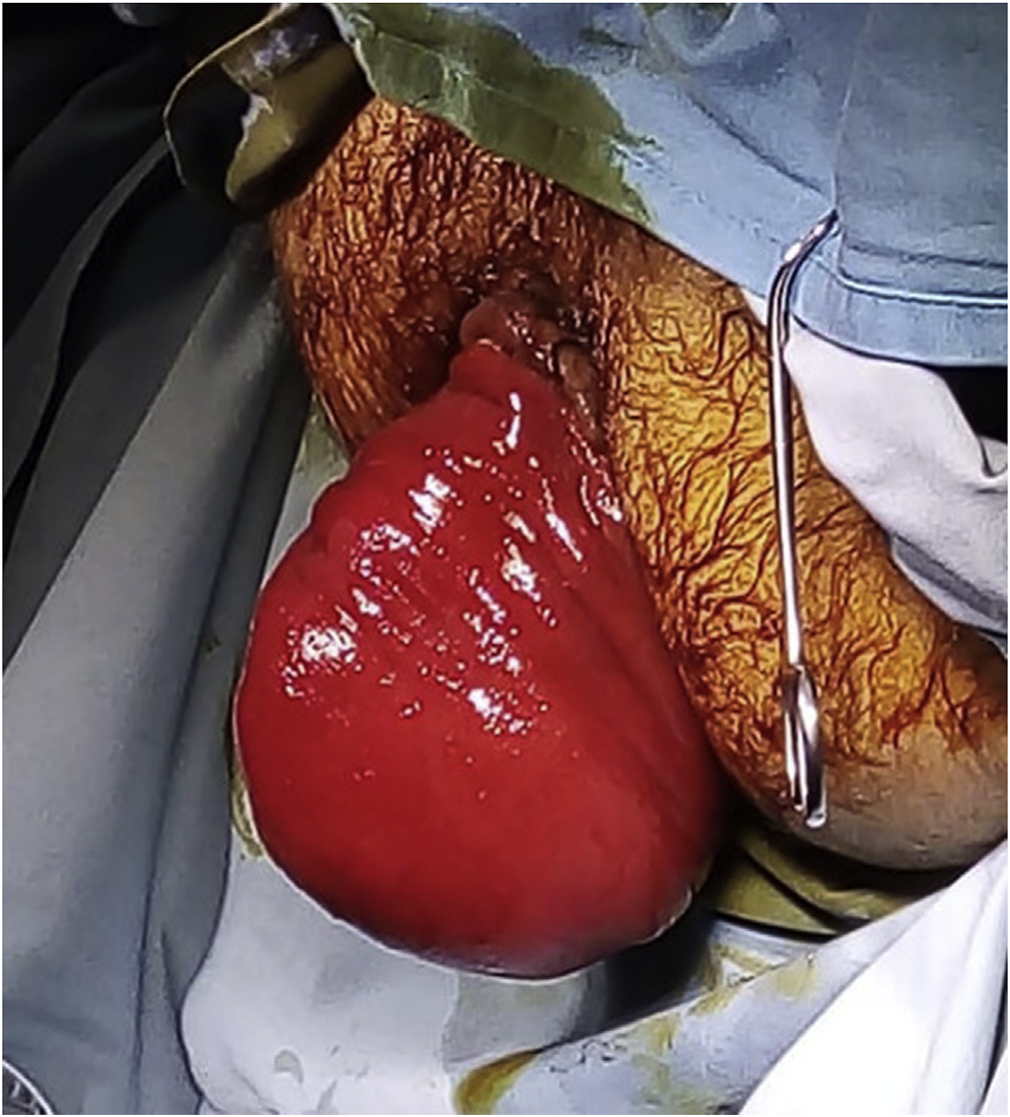
An irreducible and edematous rectal prolapse measuring 25*10 cm wide.

**Fig. 2:**
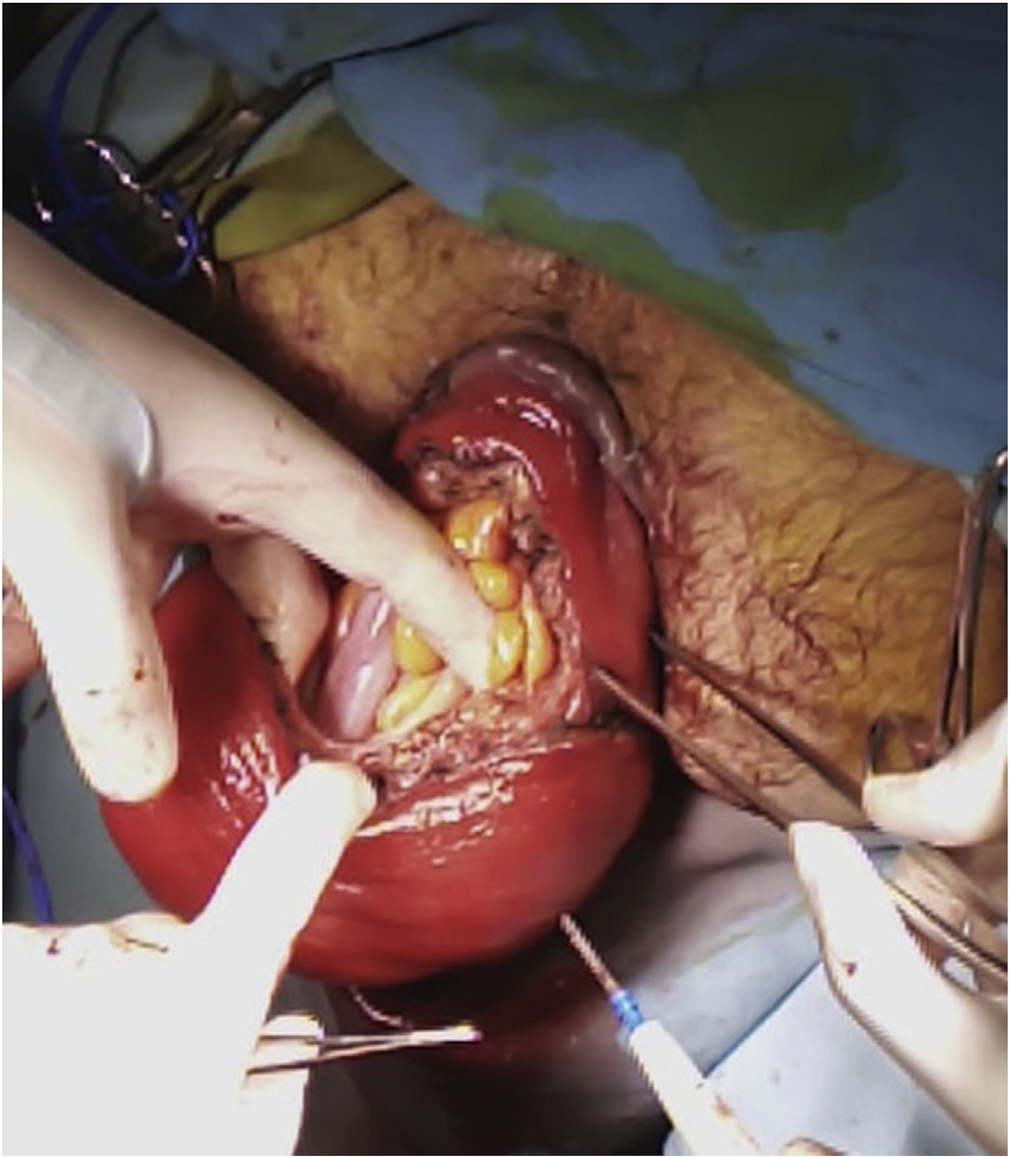
Rectum wall section just above the pectineal line.

**Fig. 3:**
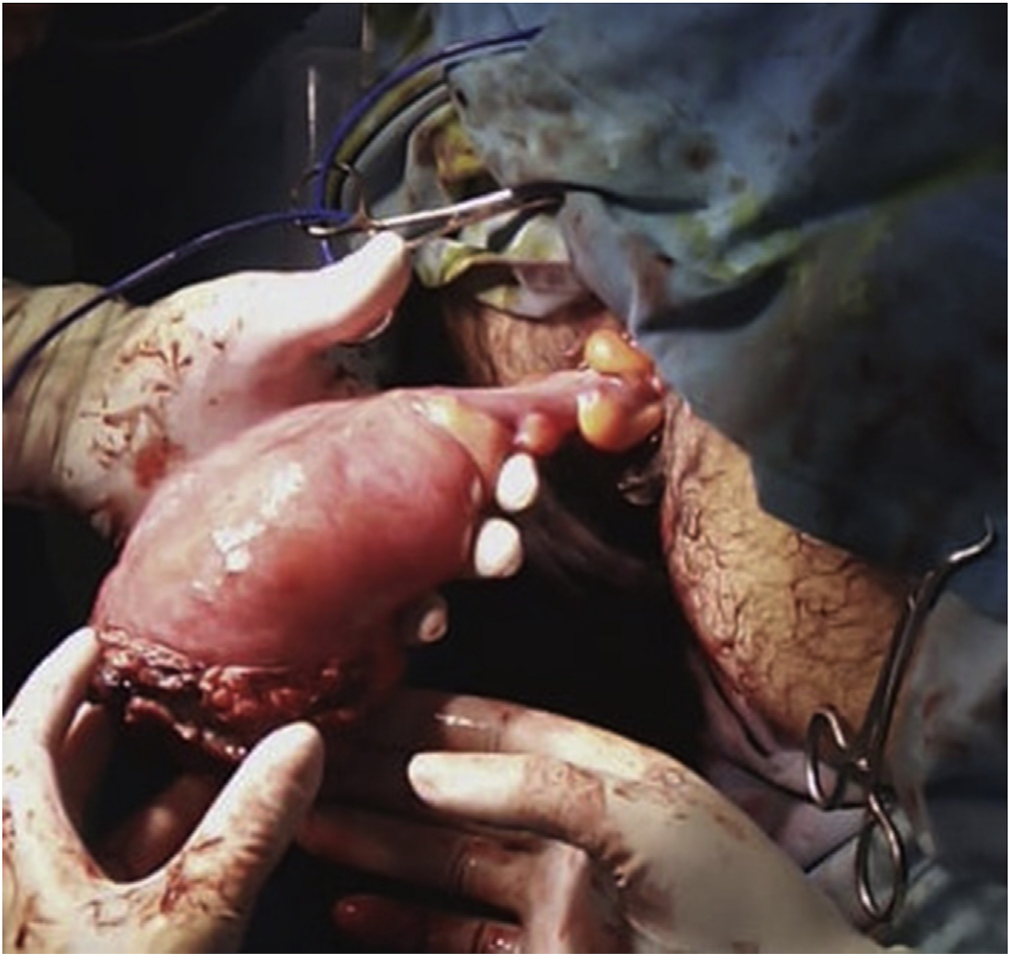
Protruded rectum.

29-year-old men, with no past medical history, consulted the Emergency Department for a sudden, painful, irreducible rectal prolapse. The patient did not report any past medical history of similar previous events, pathological defecation, or drugs use. The current history revealed multiple episodes of exteriorization reduced by digital maneuvers. Physical examination objective an afebrile patient with abdominal distention. BMI at 28 kg/m^2^. There were no signs of peritonitis. At the anus, there was a prolapse, irreducible, edematous, without signs of ischemia or necrosis measuring 25*10 cm wide (Fig. 1). The laboratory data showed a high white blood cell count (13.200/μl) and elevated C-reactive protein (75 mg/dl). After a failure of external manual reduction, the patient underwent emergent surgery. The procedure consisted of a rectosigmoidectomy with coloanal anastomosis using a perineal approach according to the Altemeier technique. The rectum was sectioned just above the pectineal line (Fig. 2). The colon was descended through the anus (Fig. 3) and was also sectioned at the level of pectineal line. The coloanal anastomosis was manually perfomed. We finished the intervention by a diverting ileostomy. The postoperative follow-up was uneventful. The patient was discharged at post-operative day five. The patient was examinated after a week in the outpoint clinic. There were no physical of biological abnormalities.

## 2 Discussion

Rectal prolapse is a relatively common disabling condition that affects women nine times more often than men [5]. In 80% of cases, it is associated with anal incontinence [5]. Its occurrence justifies surgical treatment because of its functional disorders and potential complications [6]. Strangulation of the RP is rare. It occurs in 2–4% of cases [3,4]. When the incarcerated rectum cannot be reduced, some techniques can be used to solve the situation such as sedation and the application of sucrose and salt. In case of failure or necrosis, surgical intervention becomes mandatory to restore an anatomical position of the digestive tract and enhance function. In this context of emergency, only the rectosigmoid resection using a perineal approach or the Altemeier procedure can be proposed, with or without diverting stoma [7]. The Delorme procedure is difficult in this situation given the presence of oedema and in case of necrosis becomes a counter indication [8]. The immediate postoperative morbidity for the Altemeier procedure, performed emergently, is almost inexistent with a very weak possibility of anastomotic leak [9]. In the long term, risk of recurrence remains nonetheless more elevated than of the procedures with the abdominal approach [9].

## 3 Conclusion

In this report performed according SCARE 2018 criteria [10], we could conclude that strangulated RP is a rare complication. Altemeier procedure remains the intervention of choice in this situation.

## Authors’ contributions

All authors read and approved the final manuscript.

## Funding

This work was not supported by any institution.

## Provenance and peer review

Not commissioned, externally peer reviewed.

## Author disclosures

No conflict of interest to disclose.

## Compliance with ethical standards

Consent of the patient was obtained to publish this case.
